# Prognostic factors of patients with thyroid cancer and bone metastasis at presentation

**DOI:** 10.3389/fendo.2024.1344795

**Published:** 2024-06-04

**Authors:** Zhaonong Yao, Yuhong Yao, Xiaowei Zhou, Shujia Shen, Xiaofeng Hu, Qian Gao

**Affiliations:** ^1^ Nursing Department, The Second Affiliated Hospital, Zhejiang University School of Medicine, Hangzhou, Zhejiang, China; ^2^ Department of Orthopedics, The Second Affiliated Hospital, Zhejiang University School of Medicine, Hangzhou, Zhejiang, China

**Keywords:** bone metastases, thyroid cancer, therapeutic recommendation, prognostic factors, bone cancer

## Abstract

**Objective:**

While bone metastases (BMs) are present in a minority of thyroid cancer (TC) patients at the time of initial diagnosis, there has been growing concern regarding their impact on life expectancy and quality of life. The aim of this study was to identify prognostic factors associated with overall survival (OS) and cancer-specific survival (CSS) in these patients and provide therapeutic recommendations based on the findings.

**Methods:**

In this retrospective cohort study, we included 82 patients diagnosed as TC with BM received treatment in our department from 2011.03 to 2023.03 (average follow-up duration was 3.02 years). The retrospective study was performed according to the inclusion and exclusion criteria. Kaplan-Meier analysis was used to estimate the OS and CSS, while the univariate and multivariate Cox proportional hazard models were employed to determine prognostic factors associated with OS and CSS. Also, 287 patients’ data were collected from the National Cancer Institute’s Surveillance, Epidemiology, and End Results (SEER) database between 2010 and 2015 to confirm the prognostic factors identified in the retrospective study.

**Results:**

The average survival time of the 82 patients was estimated to be 5.818 years (with a 95% confidence interval (CI) of 4.767 to 6.868 years). The cox regression analysis showed that older age (hazard ratio (HR) = 1.045, 95% CI: 1.001-1.092, P = 0.047), larger tumor size (>5cm, HR = 11.087, 95% CI: 3.728 - 32.976, P = 0.000), and the presence of extraosseous metastasis (HR = 3.247, 95% CI: 1.376 - 7.665, P = 0.007) were statistically significant factors associated with worse CSS. The results were furtherly confirmed in 287 SEER-sourced patients (age (HR = 1.020, 95% CI: 1.006 - 1.034, P = 0.006), tumor size (HR = 2.917, 95% CI: 2.044 - 4.161, P = 0.000), and extraosseous metastasis (HR = 3.726, 95% CI: 2.571 - 5.398, P = 0.000)).

**Conclusions:**

These results offer a population-based assessment of prognostic factors for patients with TC and BMs, revealing that age, primary tumor size (>5cm), and presence of extraosseous metastases are independent prognostic factors that correlate with worse survival. Accordingly, treatment for such patients ought to concentrate on systemic integrative therapy instead of surgical intervention.

## 1 Introduction

As one of the most common tumors, the prevalence of thyroid cancer (TC) increased rapidly, with a 240% increased incidence over the past three decades. While patients with localized/regional TC have a 5-year overall survival (OS) rate of 99%, those with bone metastases (BMs) have poorer 5-year OS, ranging from 41% to 79.4% (1). Bone is the second most common site for metastases in thyroid cancer, BMs can lead to the occurrence of skeletal-related events (SREs), including the need for bone irradiation and/or surgery, spinal cord compression, and pathologic fractures (2). These events are associated with a decreased quality of life and survival in TC patients (3, 4). In a previous report, BMs were found in 47% of TC patients at the time of initial diagnosis, while a subset of patients seek medical advice from orthopedist for the first time because of SREs and/or abnormal imaging findings of musculoskeletal system (5).

For TC patients experiencing bone events (BMs and SREs), treatment options such as zoledronic acid (ZA), bone irradiation, or bone surgery may be necessary (1, 6). However, it is difficult for surgeons to making the therapeutic schedule due to the lack of the prognosis outcomes. This raises three questions: (1) What is the average survival time for these patients, (2) Which epidemiological and clinical parameters can be considered as prognostic indicators, (3) Is treatment of the primary tumor necessary, and can it potentially prolong the survival time of these patients? Although comprehensive studies on cancer patients with BMs at diagnosis have been conducted, limited information is available regarding TC patients with BMs at diagnosis.

Herein, we analyzed a retrospective cohort in our department to estimate the prognostic survival outcomes in TC patients with BMs, and revealed that those patients with older age, larger tumor size (>5cm), extraosseous metastases should be were associated with worse survival time. We also verified the phenomenon in a larger population in SEER database. The surgical intervention of SREs in those patients should be carefully considered.

## 2 Methods and materials

### 2.1 Study cohort

**Figure 1:**
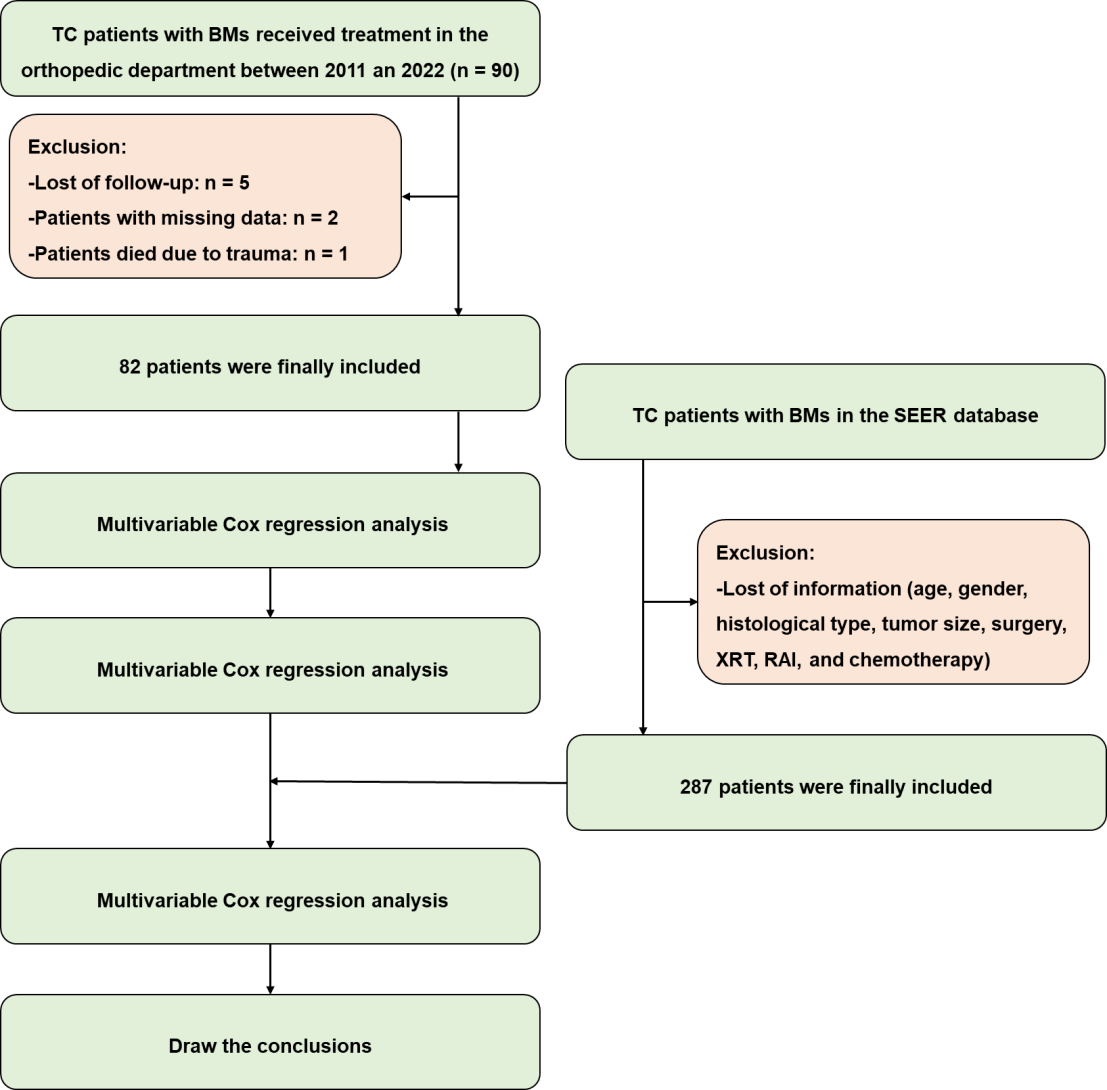
The patient inclusion and exclusion process for this study.

In this retrospective study, we conducted a thorough review of medical records for all TC patients with BMs who received treatment at the orthopedic department between March 2011 and July 2022. A total of 90 patients were included in the study after the exclusion of 5 patients due to a lack of follow-up and 2 patients due to missing information, such as age, gender, histological type, tumor size, and details of treatment including surgery, external beam radiotherapy (XRT), radioisotope (RAI), and chemotherapy. Patients with metastases to the liver, lung, or brain were also excluded. Additionally, one patient, who died due to a traffic accident, was excluded from the study. **Figure 1** illustrates the inclusion and exclusion process. The time of death or the last follow-up was recorded for each patient. Given the relatively short survival period for patients with metastatic cancers, those who died due to cancer progression were included in the study regardless of their follow-up duration. Cancer-specific survival (CSS) was determined by calculating the time from the initial diagnosis of TC to death specifically caused by cancer. Informed consent was obtained from all patients or their legal representatives, and the study was authorized by the ethics committee of our hospital (Grant No. 20230984).

The SEER database supplied us with data concerning metastatic sites labeled as “CS mets at DX-bone (2010+)”. In detail, data from SEER database was obtained as follows: the case-listing session on the SEER*Stat version 8.3.9 software was utilized to perform the analysis. In the software, we selected the primary tumor site as “thyroid”. Meanwhile, we set the SEER Combined Mets at DX-bone (2010+) to YES and finally identified the thyroid cancer patients with bone metastasis. Thus, clinical data of thyroid cancer patients with BM could be analyzed. After excluding patients who had incomplete data similar to our own study, which encompassed age, gender, histological type, tumor size, and specific treatment details like surgery, XRT, RAI, and chemotherapy, we obtained additional information from a supplementary cohort consisting of 317 patients diagnosed with TC and BMs between 2010 and 2015. This supplementary data served to bolster and reinforce our findings.

### 2.2 Statistical analysis

To determine the prognostic factors affecting patients’ survival, both univariate and multivariate Cox proportional hazard models were employed. In order to assess the statistical power of the study, a *post hoc* power analysis was conducted using G*Power 3.1.9.2. The effect size, based on the postoperative Kujala score, was calculated to be 1.14. Considering this effect size and a significance level (α) of 0.05, a statistical power exceeding 0.80 was determined.

A variable was deemed significantly associated with overall survival (OS) if its p-value was less than 0.05 in the multivariate analysis. Variables meeting this criterion were then subjected to Kaplan-Meier analysis to assess their impact on survival, and the log-rank test was employed to ascertain differences between the survival curves. All statistical analyses and survival charts were performed using SPSS 23.0 (IBM Corporation, Armonk, NY).

## 3 Results

### 3.1 Characteristics of study patients

**Figure 2:**
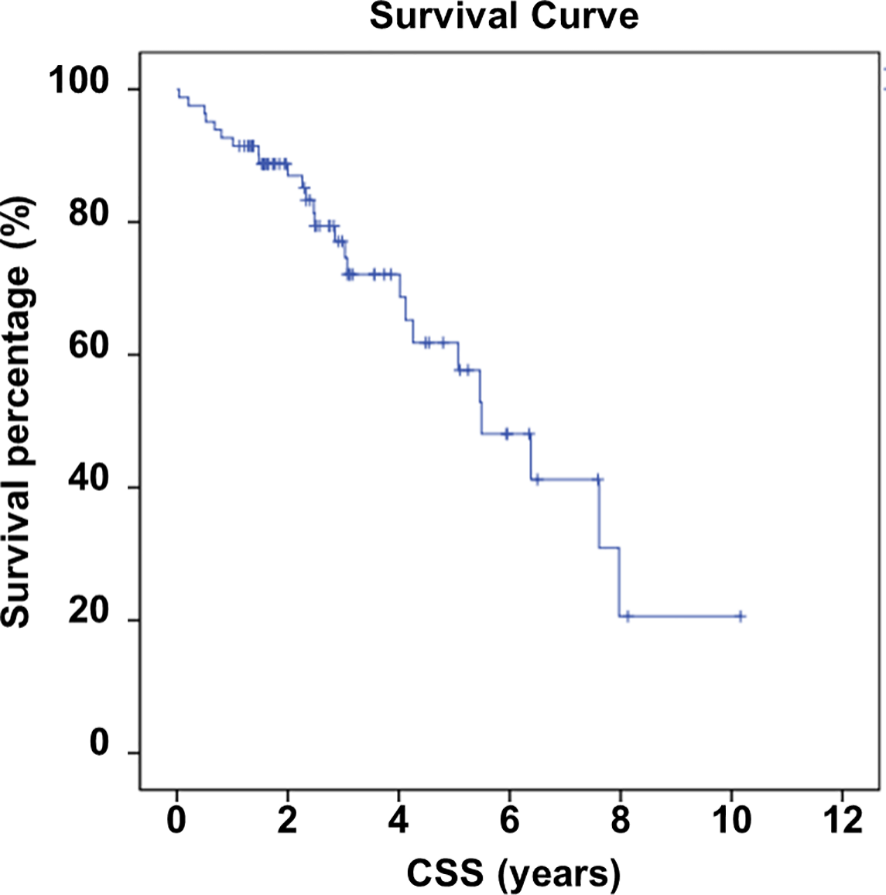
Survival of included individuals.

A total of 82 patients, with an average follow-up duration of 3.02 years (ranging from 0.02 to 10.16 years), were included in the study based on the criteria mentioned above (**Figure 1**). All patients’ information was listed in **Supplementary Table 1**, while the baseline demographic information of the patients was shown in **Table 1**. Among the included individuals, 30 were male and 52 were female, with an average age of 60.7 years (ranging from 25 to 84 years). The average time between the diagnosis of TC and the occurrence of SREs was found to be 7.12 years, ranging from 0 to 38.25 years. It is worth noting that SREs occurred within 0.1 years after the diagnosis of TC in 19 patients, indicating that the SREs might be the initial symptoms of TC. Among all patients, 64 underwent orthopedic surgery, while the remaining 18 received conservative treatment or biopsy operations. In 29 patients, TC was found to have metastasized to the lung (n = 22), liver (n = 7), or brain (n = 7, all cases combined with lung metastasis). The size of the primary lesion was also recorded, with 17 patients having lesions larger than 5 cm. As of the last follow-up, 56 patients were still alive, while 26 had succumbed to tumor progression. According to the Kaplan-Meier analysis results, the average survival time was estimated to be 5.818 years (with a 95% confidence interval of 4.767 to 6.868 years). The survival curve is depicted in **Figure 2**.

**Table 1:** Demographic characteristics of 82 patients in the retrospective cohort.

Indexes	Value
Age	60.7 (25 - 84)
Gender (Male/Female)	30/52
Follow-up duration (years)	3.02 (0.02 - 10.16)
CSS (years)	5.818 (0.04 – 10.16)
Survive (Y/N)	56/26
Bone metastasis duration (years)	7.12 (0 – 38.25)
Other metastases (Y/N)	29/53
Tumor size > 5 cm (Y/N)	17/65
Thyroid surgery	74/8
XRT	11/71
RAI	54/28
Histological types (FTC/PTC/MTC)	67/11/4

FTC, Follicular Thyroid Carcinoma; MTC, Medullary Thyroid Carcinoma; PTC, Papillary Thyroid Carcinoma; RAI, Radioisotope; XRT, External Beam Radiotherapy.

### 3.2 Prognostic factors for OS of patients with BMs in the retrospective cohort

We conducted multivariable Cox regression analysis to identify potential factors associated with prognostic outcomes. The collinearity diagnostics results revealed no correlation among the independent variables (see **Supplementary Table 2**), indicated by a VIF < 10. These variables included sex, age, tumor size, histological type, absence of thyroid surgery, XRT, and RAI. The results indicated that older age (hazard ratio (HR) = 1.045, 95% confidence interval (CI): 1.001-1.092, P = 0.047), larger tumor size (>5cm, HR = 11.087, 95% CI: 3.728 - 32.976, P = 0.000), and the presence of extraosseous metastasis (HR = 3.247, 95% CI: 1.376 - 7.665, P = 0.007) were statistically significant factors associated with worse CSS (refer to **Table 2**).

**Table 2:** Multivariable COX regression analysis results for CSS in TC patients with BMs.

	HR	P value	CI
Age	1.045	**0.047**	1.001 – 1.092
Size	0.094	**0.000**	0.030 – 0.268
Metastasis	0.309	**0.007**	0.130 – 0.727
Sex	1.585	0.250	–
Histological types (FTC as control)			–
Histological types (PTC)	0.204	0.707	–
Histological types (MTC)	0.482	0.474	–
Thyroid surgery	2.279	0.145	–
RAI	0.004	0.924	–
XRT	1.133	0.295	–

RAI, Radioisotope; XRT, External Beam Radiotherapy,

Values in bold mean statistical significant.

**Figure 3:**
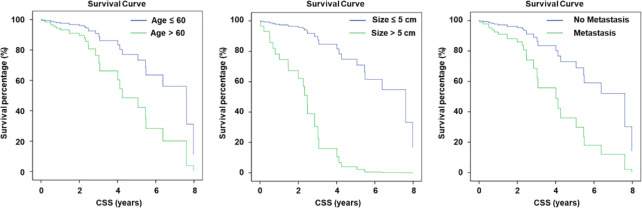
Ages of patients, tumor size, and metastasis status are prognostic factors for included patients.

Furthermore, the univariate Cox regression analysis further demonstrated that age, tumor size (>5cm), and extraosseous metastasis were statistically significant and associated with worse CSS. The statistical results are presented in **Table 3**. We additionally confirmed that age (HR = 1.054, 95% CI: 1.011 - 1.098, P = 0.014), tumor size (HR = 9.002, 95% CI: 3.100 - 26.141, P = 0.000), and extraosseous metastasis (HR = 3.066, 95% CI: 1.340 - 7.016, P = 0.008) were associated with CSS outcomes and could be considered as prognostic factors. The survival curve for these three factors is presented in **Figure 3**.

**Table 3:** Univariate COX regression analysis results for CSS in TC patients with BMs.

	HR	P value	CI
Age	1.054	**0.014**	1.011 – 1.098
Size	9.002	**0.000**	3.100 – 26.141
Metastasis	3.066	**0.008**	1.34 – 7.016

Values in bold mean statistical significant.

### 3.3 The verification of the prognostic factors in TC patients with BMs in SEER database

**Figure 4:**
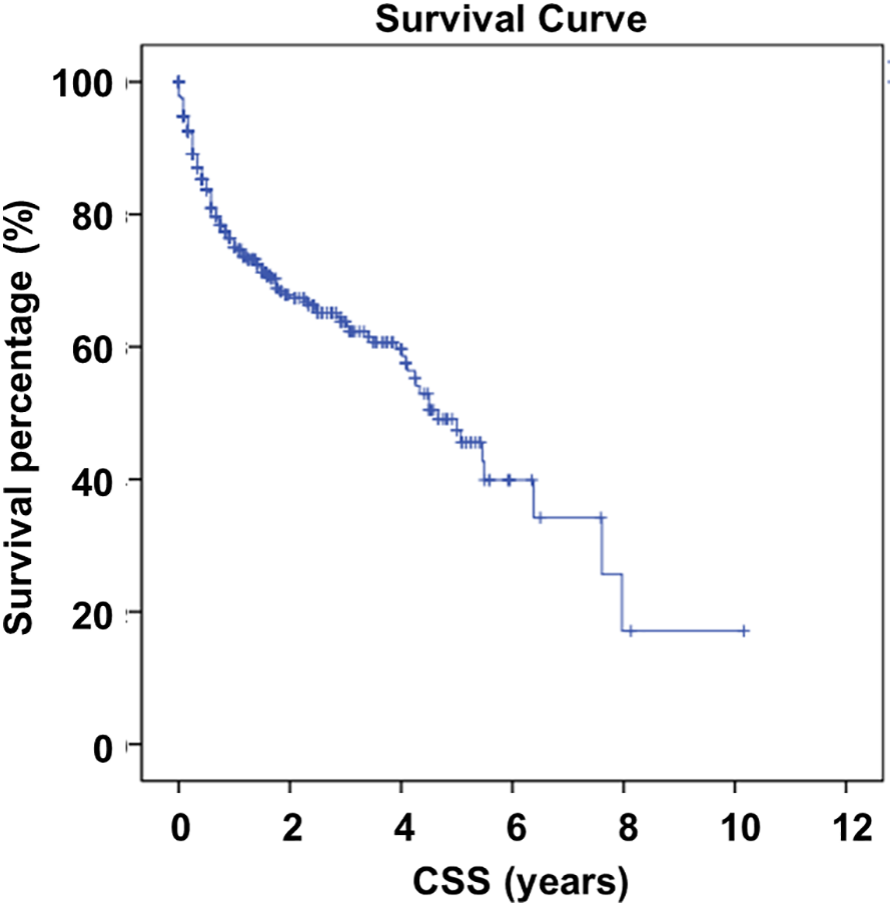
External validation using SEER database of patient survival.

A total of 287 patients were included in the analysis based on the criteria mentioned above. The distribution of patient characteristics is summarized in **Table 4**. The average age was 64.68 years old (ranging from 20 to 106 years), and there were 136 males and 151 females included in the study. The CSS was 4.831 years (ranging from 0 to 5.92 years), and extraosseous metastasis was present in 243 patients. Among 175 patients, the tumor size was over 5 cm. We combined the data from the database with our retrospective data, and the survival curve is shown in **Figure 4**.

**Table 4:** Demographic and clinical characteristics of 287 patients with TC and BMs at diagnosis.

Indexes	Value
Age	64.9 (20 - 106)
Gender (Male/Female)	136/151
CSS (years)	4.831 (0 – 5.92)
Survive (Y/N)	184/103
Other metastases (Y/N)	243/44
Tumor size > 5 cm (Y/N)	175/112

**Figure 5:**
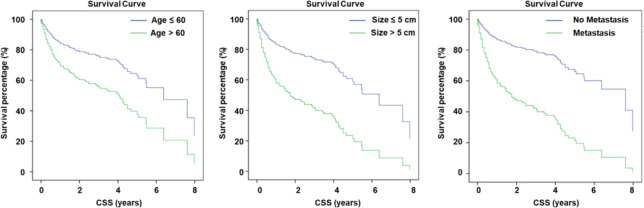
Data from SEER database revealed similar results of prognostic factors for TC patients.

Next, we performed univariate COX regression analysis to identify whether the examined factors could serve as prognostic factors in TC patients with BMs. The results indicated that age (HR = 1.020, 95% CI: 1.006 - 1.034, P = 0.006), tumor size (HR = 2.917, 95% CI: 2.044 - 4.161, P = 0.000), and extraosseous metastasis (HR = 3.726, 95% CI: 2.571 - 5.398, P = 0.000) were associated with CSS outcomes and could be considered as prognostic factors (**Table 5**). The survival curves for these three factors are presented in **Figure 5**.

**Table 5:** Univariate Cox regression analysis of variables in TC patients with BMs.

	HR	P value	CI
**Age**	1.020	0.006	1.006 – 1.034
**Size**	2.917	0.000	2.044 – 4.161
**Metastasis**	3.726	0.000	2.571 – 5.398

## 4 Discussion

BMs are an uncommon but serious complication of aggressive TC. Previous studies aimed at identifying factors that influenced the overall and cancer-specific mortality of TC patients with BMs (BMs were present either at their first diagnosis or during the follow-up period). Herein, we first used a retrospective cohort to seek for the risk factors of the worse survival outcomes, and following verified the results in a multiple-center SEER database between 2010 to 2015. In patients with BMs at diagnosis of prostate cancer (7), renal cell carcinoma (8), bladder cancer (9), and lung cancer (10), the survival time was 20, 12, 4, 3 months, respectively. In our work, the average survival time was much longer, which was estimated to be 5.818 years and 4.831 years separately in the retrospective cohort and database information. Combined with the previous results that the 5-year survival for patients with BMs from TC was 79.4%, the longer survival time indicated that the management of SREs in TC patients with BM is of great value, and our results may provide some references for doctors when giving treatment recommendations to these patients.

Multivariate analysis identified three prognostic factors in patients with TC and BMs correlated with worse CSS, including age, tumor size (>5cm), and presence of extraosseous metastases. The factors were verified in the following univariate COX regression analysis. However, the HR of age factor is too low to predict the worse outcome event, although the difference is of statically significance. The other important aspect is the extraosseous metastases. As is known, in the early stage of thyroid cancer, cervical lymph nodes may metastasize or invade the surrounding thyroid tissues, such as trachea, esophagus, recurrent laryngeal nerve, etc. With the development of the tumor, it can be transferred to the lungs, bones and other organs through blood transport. The treatment of those patients should focus on the systemic integrative therapy instead of the surgical intervention, while the management of SREs should be more conservative. Previously, Anastassios et al. (5) found that papillary, follicular, poorly differentiated, and undifferentiated histological types, were all adverse prognostic factors for patients’ survival when compared to papillary in TC patients with BMs. However, in our study, the histological types of the TC had no statistical significance in the prognosis. Instead, the tumor size of primary TC is tightly linked with the prognosis. Herein, we simply divided the tumor size with the scale of 5 cm, and found that those patients with larger tumor size have a less CSS, with the HR of 2.917. This phenomenon indicated the orthopedists that the focus on the primary lesion is of great significance, which cannot be neglected.

When it turned to the treatment of the primary lesion, we found that no matter XRT, RAI, and surgical intervention showed no significant difference in the outcome events. In the SEER database, a great number of patients (39.7%) in TC patients with BMs at diagnosis did not receive surgical treatment of primary tumor, possibly because of the occurrence of BMs, while previous studies usually did not consider the surgical treatment for primary tumor as a prognostic factor to analyze, as most of patients with BMs received total or near total thyroidectomy (5, 11, 12). XRT was not a significant factor as well in both patients with BMs and patients with BMs only by multiple analysis. The same results were observed in TC patients with BMs by Slook et al. (12) and Bernier et al. (11). Patients whose metastatic site had RAI uptake usually received RAI to the maximum permissible dose (13). A study showed no impact of RAI on survival (5), whereas other researchers found such therapy had valuable effect on TC patients with BMs (11, 13, 14). These results are consistent with our present work. Of note, management of vertebral bone lesions, including both primary tumor or metastatic tumor, is the key to reach a favored outcome of certain patients (15). Surgical strategies including en bloc resection, which shares a similar principle with intervertebral disc replacement (16), might help in improving patient prognosis, but further studies especially prospective research is urgently needed.

Inevitably, there are some limitations in the present study. First, the sample size, i.e. 82 patients, is still small in the work, and the follow-ups was not very long, although the *post hoc* analysis was used to determine the power of analysis. The selection bias was not avoided as all of the data was obtained from database of hospitals. Second, information on the number, location and management of bone lesions was unavailable in SEER database and limited our precise analysis. So, we just used the data from SEER to confirm our results in the retrospective study. Third, actual incidence of BMs in TC patients at diagnosis might be underestimated because some asymptomatic patients might not be recorded in, which might cause the underestimation of the importance of those prognostic factors.

## 5 Conclusion

In conclusion, age, tumor size (>5cm), and presence of extraosseous metastases were present as independent prognostic factors associated with worse survival in patients with TC and BMs. The treatment of those patients should focus on the systemic integrative therapy, while the management of SREs should be more conservative.

## Data availability statement

The original contributions presented in the study are included in the article/**Supplementary Material**. Further inquiries can be directed to the corresponding author.

## Ethics statement

The studies involving humans were approved by The IRB review bord of The Second Affiliated Hospital of Zhejiang University School of Medicine. The studies were conducted in accordance with the local legislation and institutional requirements. Written informed consent for participation in this study was provided by the participants’ legal guardians/next of kin.

## Author contributions

ZY: Conceptualization, Investigation, Writing – original draft. YY: Data curation, Methodology, Writing – original draft. XZ: Writing – original draft. SS: Formal Analysis, Writing – original draft. XH: Investigation, Resources, Writing – original draft. QG: Writing – original draft, Writing – review & editing.
