# Supplementary material for: Prognostic factors of patients with thyroid cancer and bone metastasis at presentation
Source: Front Endocrinol (Lausanne). 2024 Jun 4;15:1344795. doi: 10.3389/fendo.2024.1344795 (PMC11186379; doi:10.3389/fendo.2024.1344795)
Supplement: Supplementary file 1 [file Table_1.docx]

**s.Table 1 The information of patients in the retrospective cohort.**

| Patients | Sex | Age | Follow-up duration | Survive | CCS (years) | Histological type | Bone metastasis duration | Other metastasis | Tumor size | Thyroid surgery | RAI | XRT |
| --- | --- | --- | --- | --- | --- | --- | --- | --- | --- | --- | --- | --- |
| 1 | M | 62 | 10.16 | Y | 10.16 | PTC | 2.62 | N | Y | N | Y | N |
| 2 | F | 59 | 8.13 | Y | 8.13 | FTC | 0.06 | N | Y | Y | Y | N |
| 3 | M | 40 | 7.96 | N | 7.97 | FTC | 0.08 | N | Y | Y | Y | N |
| 4 | M | 68 | 7.61 | N | 7.61 | FTC | 0.05 | N | Y | Y | N | N |
| 5 | M | 48 | 7.59 | Y | 7.59 | FTC | 3.19 | N | Y | Y | Y | N |
| 6 | F | 77 | 6.50 | Y | 6.50 | FTC | 10.28 | Lu | Y | Y | N | N |
| 7 | F | 68 | 6.34 | N | 6.38 | FTC | 30.39 | Lu | Y | Y | N | Y |
| 8 | F | 53 | 6.35 | Y | 6.35 | FTC | 15.44 | Lu | Y | Y | Y | N |
| 9 | F | 26 | 5.96 | Y | 5.96 | FTC | 1.82 | N | Y | Y | Y | N |
| 10 | F | 54 | 5.94 | Y | 5.94 | FTC | 11.85 | N | Y | Y | Y | N |
| 11 | F | 53 | 5.48 | N | 5.49 | FTC | 2.52 | Lu | Y | Y | Y | N |
| 12 | F | 64 | 5.42 | N | 5.46 | FTC | 6.35 | Lu | Y | Y | Y | N |
| 13 | M | 54 | 5.00 | N | 5.07 | FTC | 0.01 | Lu + Li | Y | Y | Y | N |
| 14 | F | 66 | 5.25 | Y | 5.25 | FTC | 6.53 | N | Y | Y | Y | N |
| 15 | F | 66 | 5.10 | Y | 5.10 | PTC | 0.01 | N | Y | Y | Y | N |
| 16 | F | 52 | 4.80 | Y | 4.80 | FTC | 5.98 | N | Y | Y | Y | N |
| 17 | F | 43 | 4.54 | Y | 4.54 | FTC | 0.07 | N | Y | Y | N | N |
| 18 | M | 50 | 4.48 | Y | 4.48 | FTC | 0.38 | N | Y | Y | Y | N |
| 19 | F | 64 | 4.11 | N | 4.26 | MTC | 9.39 | Lu + Li | Y | Y | N | N |
| 20 | F | 63 | 4.05 | N | 4.12 | FTC | 4.86 | Lu + Br | Y | Y | N | N |
| 21 | F | 41 | 3.86 | Y | 3.86 | FTC | 1.42 | N | Y | Y | Y | N |
| 22 | M | 55 | 3.74 | Y | 3.74 | PTC | 10.04 | N | Y | Y | Y | Y |
| 23 | M | 58 | 3.56 | Y | 3.56 | FTC | 12.22 | N | Y | Y | Y | N |
| 24 | F | 25 | 3.56 | Y | 3.56 | PTC | 0.64 | N | Y | Y | Y | Y |
| 25 | F | 73 | 4.01 | N | 4.02 | PTC | 0.04 | N | Y | Y | Y | N |
| 26 | F | 71 | 3.02 | N | 3.03 | FTC | 7.20 | Lu | Y | Y | Y | Y |
| 27 | M | 63 | 3.00 | N | 3.07 | FTC | 0.00 | Lu + Li | Y | N | N | N |
| 28 | F | 55 | 3.17 | Y | 3.17 | PTC | 0.11 | N | Y | Y | Y | N |
| 29 | M | 58 | 3.12 | Y | 3.12 | FTC | 10.66 | N | Y | Y | N | N |
| 30 | F | 53 | 3.12 | Y | 3.12 | FTC | 12.67 | Lu | Y | Y | Y | Y |
| 31 | F | 63 | 3.07 | Y | 3.07 | FTC | 20.72 | N | Y | Y | Y | N |
| 32 | M | 55 | 2.98 | Y | 2.98 | PTC | 0.05 | N | Y | Y | Y | N |
| 33 | M | 51 | 2.75 | N | 2.85 | FTC | 0.02 | Lu + Li | N | Y | N | Y |
| 34 | F | 53 | 2.91 | Y | 2.91 | FTC | 0.03 | N | Y | Y | Y | N |
| 35 | F | 70 | 2.76 | Y | 2.76 | FTC | 6.01 | N | Y | Y | N | N |
| 36 | F | 61 | 2.75 | Y | 2.75 | FTC | 20.03 | N | N | Y | Y | N |
| 37 | F | 52 | 2.74 | Y | 2.74 | FTC | 10.04 | N | Y | Y | Y | N |
| 38 | M | 52 | 2.49 | N | 2.47 | FTC | 3.64 | N | Y | Y | N | Y |
| 39 | F | 69 | 2.57 | Y | 2.57 | FTC | 19.21 | Lu | Y | Y | Y | N |
| 40 | F | 65 | 2.28 | N | 2.26 | MTC | 15.99 | Lu | N | Y | N | N |
| 41 | F | 69 | 2.51 | Y | 2.51 | FTC | 13.27 | N | Y | Y | Y | N |
| 42 | F | 75 | 2.49 | Y | 2.49 | FTC | 0.04 | Lu | Y | Y | Y | N |
| 43 | F | 59 | 2.24 | N | 2.33 | FTC | 0.08 | N | N | Y | Y | N |
| 44 | F | 45 | 2.40 | Y | 2.40 | PTC | 4.38 | Lu | Y | Y | Y | N |
| 45 | F | 67 | 2.33 | Y | 2.33 | FTC | 0.03 | N | N | Y | N | N |
| 46 | M | 67 | 1.99 | N | 2.00 | FTC | 0.02 | N | Y | Y | Y | N |
| 47 | M | 60 | 1.97 | Y | 1.97 | FTC | 0.06 | Lu | N | N | N | N |
| 48 | F | 57 | 1.94 | Y | 1.94 | FTC | 20.85 | N | Y | Y | Y | N |
| 49 | M | 66 | 1.41 | N | 1.47 | FTC | 3.83 | Lu | Y | Y | Y | Y |
| 50 | F | 75 | 1.85 | Y | 1.85 | FTC | 21.93 | Lu | Y | Y | N | N |
| 51 | M | 63 | 1.78 | Y | 1.78 | FTC | 1.99 | N | Y | Y | Y | Y |
| 52 | M | 71 | 1.74 | Y | 1.74 | FTC | 9.04 | N | Y | Y | Y | N |
| 53 | F | 74 | 1.65 | Y | 1.65 | FTC | 11.13 | Lu | Y | Y | N | N |
| 54 | F | 62 | 1.64 | Y | 1.64 | FTC | 15.14 | N | Y | Y | Y | N |
| 55 | F | 68 | 1.62 | Y | 1.62 | FTC | 20.16 | N | Y | Y | Y | N |
| 56 | F | 57 | 1.45 | N | 1.48 | FTC | 9.50 | Lu | Y | Y | Y | Y |
| 57 | F | 60 | 1.58 | Y | 1.58 | FTC | 30.22 | N | Y | Y | Y | N |
| 58 | M | 64 | 1.58 | Y | 1.58 | FTC | 11.21 | N | N | Y | Y | N |
| 59 | M | 53 | 1.57 | Y | 1.57 | FTC | 0.12 | Lu | Y | Y | Y | N |
| 60 | F | 75 | 1.56 | Y | 1.56 | FTC | 2.21 | N | Y | Y | N | N |
| 61 | F | 60 | 1.55 | Y | 1.55 | FTC | 38.25 | Lu | Y | Y | Y | N |
| 62 | F | 55 | 1.38 | Y | 1.38 | FTC | 0.07 | N | Y | Y | Y | N |
| 63 | F | 52 | 1.37 | Y | 1.37 | PTC | 0.99 | N | Y | Y | Y | N |
| 64 | F | 80 | 1.34 | Y | 1.34 | FTC | 6.44 | Lu | Y | Y | Y | N |
| 65 | F | 58 | 1.32 | Y | 1.32 | FTC | 0.13 | N | Y | Y | Y | N |
| 66 | M | 64 | 1.28 | Y | 1.28 | FTC | 0.08 | Lu | Y | Y | Y | N |
| 67 | F | 55 | 1.22 | Y | 1.22 | FTC | 0.05 | N | Y | Y | Y | N |
| 68 | M | 58 | 1.13 | Y | 1.13 | FTC | 9.65 | Lu | Y | Y | Y | N |
| 69 | M | 44 | 1.01 | N | 1.01 | FTC | 5.15 | Lu | Y | Y | Y | N |
| 70 | M | 74 | 0.75 | N | 0.80 | FTC | 1.00 | N | N | Y | N | N |
| 71 | M | 62 | 0.66 | N | 0.68 | MTC | 30.76 | Lu + Li | Y | Y | N | N |
| 72 | F | 78 | 0.02 | N | 0.04 | FTC | 0.23 | N | N | N | N | N |
| 73 | F | 48 | 1.73 | Y | 1.73 | FTC | 1.04 | N | N | N | N | N |
| 74 | F | 75 | 0.49 | N | 0.50 | FTC | 0.66 | N | N | N | N | N |
| 75 | M | 75 | 1.63 | Y | 1.63 | FTC | 12.15 | N | N | N | N | N |
| 76 | F | 76 | 1.57 | Y | 1.57 | FTC | 19.22 | N | Y | Y | N | N |
| 77 | M | 68 | 1.53 | Y | 1.53 | FTC | 14.25 | N | Y | Y | Y | Y |
| 78 | F | 84 | 0.17 | N | 0.21 | FTC | 0.15 | N | N | N | N | N |
| 79 | M | 67 | 2.48 | N | 2.48 | MTC | 1.30 | N | N | Y | Y | N |
| 80 | M | 45 | 2.83 | Y | 2.83 | PTC | 2.94 | N | N | Y | N | N |
| 81 | M | 72 | 2.30 | Y | 2.30 | PTC | 1.06 | N | N | Y | N | N |
| 82 | F | 67 | 0.47 | N | 0.52 | FTC | 0.49 | Lu + Li | N | Y | N | N |

Note: F: female, M: male, Y: yes, N: no, Lu: lung, Li: liver, Br: brain, FTC: Follicular Thyroid Carcinoma, MTC: Medullary Thyroid Carcinoma, PTC: Papillary Thyroid Carcinoma, RAI: Radioisotope, XRT: External Beam Radiotherapy.

**s.Table2 The diagnosis of collinearity in the retrospective cohort**

| **Indexes** | **Tolerance** | **VIF** |
| --- | --- | --- |
| Gender | 0.953 | 1.049 |
| Age | 0.835 | 1.197 |
| Histological types | 0.864 | 1.158 |
| Size | 0.650 | 1.538 |
| Thyroid surgery | 0.700 | 1.429 |
| RAI | 0.663 | 1.509 |
| XRT | 0.913 | 1.096 |
| Metastasis | 0.848 | 1.180 |

Notes: The indexes with tolerance > 0.1, VIF<5 were diagnosed without collinearity.

RAI: Radioisotope, XRT: External Beam Radiotherapy, VIF: Variance Inflation Factor.
